# Rev. Henry Edwin Scott

**Published:** 1911-04-22

**Authors:** 


News has been received of the death at Kikuyu, British East Africa, of the Rev. Henry Edwin Scott, L.R.C.P. and S.E., Church of Scotland medical missionary, in his forty-eighth year. Mr. Scott studied at Edinburgh University, and had been a missionary since 1890. He was also a good athlete. The *Times* recalls that after serving in Nyasaland, in December 1907 he was transferred to Kikuyu as the head of the Church of Scotland Mission there. He was a member of the Government Board of Education, and was often consulted by the Government about native affairs.

